# An Assessment of Selected Nutritional, Bioactive, Thermal and Technological Properties of Brown and Red Irish Seaweed Species

**DOI:** 10.3390/foods10112784

**Published:** 2021-11-12

**Authors:** Halimah O. Mohammed, Michael N. O’Grady, Maurice G. O’Sullivan, Ruth M. Hamill, Kieran N. Kilcawley, Joseph P. Kerry

**Affiliations:** 1 Food Packaging, School of Food and Nutritional Sciences, College of Science, Engineering and Food Science, University College Cork, T12 K8AF Cork, Ireland; halimahmoh8@gmail.com (H.O.M.); Michael.OGrady@ucc.ie (M.N.O.); 2 Sensory Groups, School of Food and Nutritional Sciences, College of Science, Engineering and Food Science, University College Cork, T12 K8AF Cork, Ireland; 3 Food Quality and Sensory Science Department, Teagasc Food Research Centre, Ashtown, D15 DY05 Dublin, Ireland; ruth.hamill@teagasc.ie; 4 Food Quality and Sensory Science Department, Teagasc Food Research Centre, Moorepark, Fermoy, P61 P996 Cork, Ireland; Kieran.kilcawley@teagasc.ie

**Keywords:** seaweeds, functional ingredient, nutritional composition, dietary fibre, antioxidant capacity

## Abstract

Irish edible brown (*Himanthalia elongata*—sea spaghetti, *Alaria esculenta*—Irish wakame) and red seaweeds (*Palmaria palmata*—dulse, *Porphyra umbilicalis*—nori) were assessed for nutritional (proximate composition; salt; pH; amino acid; mineral and dietary fibre contents); bioactive (total phenolic content (TPC) and in vitro antioxidant activity (DPPH and FRAP)); thermal (thermogravimetric analysis (TGA)); and technological (water holding capacity (WHC), oil holding capacity (OHC) and swelling capacity (SC)) properties. Red seaweeds had higher (*p* < 0.05) protein levels, whereas brown seaweeds possessed higher (*p* < 0.05) moisture, ash, insoluble and total dietary fibre contents. Nori had the lowest (*p* < 0.05) salt level. Seaweed fat levels ranged from 1 to 2% DW. Aspartic and glutamic acids were the most abundant amino acids. The total amino acid (TAA) content ranged from 4.44 to 31.80%. Seaweeds contained numerous macro (e.g., Na) and trace minerals. The TPC, DPPH and FRAP activities followed the order: sea spaghetti ≥ nori > Irish wakame > dulse (*p* < 0.05). TGA indicated maximum weight loss at 250 °C. Dulse had the lowest (*p* < 0.05) WHC and SC properties. Dulse and nori had higher (*p* < 0.05) OHC than the brown seaweeds. Results demonstrate the potential of seaweeds as functional food product ingredients.

## 1. Introduction

Seaweeds (marine macroalgae) are divided into three significant phyla or by colour: Phaeophyta (brown algae), Rhodophyta (red algae) and Chlorophyta (green algae). Since ancient times, macroalgae have been consumed widely as food in East Asia (mainly in Japan, China and Korea) [1]. Seaweeds can be classified as functional foods due to their unique nutritional and chemical properties. Epidemiological studies attribute to regular consumption of seaweeds several health benefits related to cardiovascular disease, weight management, cancer, metabolic syndrome, type 2 diabetes, digestive tract health and bone health [2].

Bioactive compounds present in seaweeds include polyphenols (phloroglucinol, phenolic acids and flavonoids), polysaccharides, pigments (carotenoids such as fucoxanthin and β-carotene), proteins (phycobiliproteins), peptides, α-tocopherol (vitamin E) and ascorbic acid (vitamin C) [3,4,5]. Bioactive properties associated with these compounds are related to antioxidant, antibacterial, antiviral and antifungal activities [6]. Nutritionally, seaweed polysaccharides are a potential source of total dietary fibre (TDF). The TDF content of seaweeds is classified into soluble and insoluble fibre fractions. TDF is made up of a range of structural and storage polysaccharides, and intercellular mucilage.

Several edible brown (e.g., *Ascophyllum nodusum*—Egg Wrack, *Himanthalia elongata*—sea spaghetti and *Alaria esculenta*—Irish wakame) and red (e.g., *Palmaria palmata*—dulse, *Porphyra umbilicalis*—nori and *Chondrus crispus*—Irish moss) seaweed species are currently available on the Irish market in dried (whole or milled) form. Seaweeds consumed as foods are often referred to as ‘sea vegetables’. Sea spaghetti has a very mild taste and flavour when soaked and consumed as a salad ingredient. Irish wakame (*Alaria esculenta*), also known as ‘dabberlocks’, is commonly served either as a vegetable or as salad leaves. The colour of brown seaweed is mainly due to the presence of fucoxanthin, an orange carotenoid, which has been reported to possess antimicrobial and antioxidant properties [7]. Polysaccharides in brown seaweeds (alginate, laminaran, sulphated fucans (fucoidan), cellulose and mannitol) represent up to 84% of the total dry weight [8]. The soluble dietary fibre (SDF) fraction in brown seaweeds is comprised of laminarin, fucoidan and alginate; and the insoluble dietary fibre (IDF) is made of cellulose. Other components of the IDF fraction in brown seaweeds are uronic acids from alginate and neutral sugars [9,10].

The red seaweed dulse (*Palmaria palmata*) is one of the most widely used and consumed seaweed species in Ireland and is generally eaten dried and uncooked. Nori (*Porphyra umbilicalis*) is usually deep red to purple in colour and has a lettuce-like appearance. Red phycobilin pigments (phycoerythrin and phycocyanin) mask the green pigments chlorophyll-a and beta-carotene and are responsible for the colour of red seaweed species [11]. Polysaccharides (SDF) present in red seaweeds are sulphated galactans (agar and carrageenans), representing approximately 70% of the cell wall constituents. The remaining polysaccharides (IDF) are composed of cellulose (7–24%) [12]. However, the IDF fraction of nori contains insoluble mannan and xylan.

From a technological perspective, brown and red seaweed types have been incorporated into foods to improve nutritional quality and food product functionality. For example; the influence of *Fucus vesiculosus* on the physical and textural properties of wheat bread [13]; the fortification of *Eucheuma cottonii* on the nutritional properties, iodine content and glycaemic index of pasta [14]; *Kappaphycus alvarezii* on the physico-chemical properties of muffins [15]; and *Himanthalia elongata* (sea spaghetti), *Undaria pinnatifida* (wakame), *Palmaria palmata* (dulse) and *Porphyra umbilicalis* (nori) on reduced salt frankfurters [16].

A deeper knowledge of the unique compositional and nutritional properties associated with Irish seaweeds would benefit food producers and consumers alike. Therefore, the objective of this study was to assess the suitability of brown (sea spaghetti and Irish wakame) and red (dulse and nori) seaweed species harvested from the coast of Ireland as potential health-promoting techno-functional ingredients for use in food products. Seaweeds were characterised based on their nutritional, bioactive (antioxidant), thermogravimetric and technological properties.

## 2. Materials and Methods

### 2.1. Reagents and Chemicals

Folin–Ciocalteu reagent, sodium carbonate (Na_2_CO_3_), gallic acid ((HO)_3_C_6_H_2_CO_2_H), 2,2-diphenyl-1-picrylhydrazyl (DPPH), (±)-6-hydroxy-2,5,7,8-tetramethylchromane-2-carboxylic acid (Trolox) (C_14_H_18_O_4_), sodium chloride (NaCl), acetone (CH_3_COCH_3_), hydrogen peroxide (H_2_O_2_), boric acid (H_3_BO_3_), hydrochloric acid (HCl), chloroform (CHCl_3_), sulphuric acid (H_2_SO_4_), methanol (CH_3_OH), 2,4,6-tris(2-pyridyl)-*s*-triazine (TPTZ) (C_18_H_12_N_6_), ethanol (CH_3_CH_2_OH), glacial acetic acid (CH_3_CO_2_H), sodium acetate trihydrate (CH_3_COONa·3H_2_O) and sodium azide (NaN_3_) were supplied by Sigma—Aldrich Ireland Ltd., Vale Road, Arklow, Wicklow, Ireland. A dietary fibre enzyme kit, β-glucan (barley), casein and high-amylose maise starch, were purchased from Megazyme, Bray, Co., Wicklow, Ireland. Silver nitrate and potassium chromate (K_2_CrO_4_) were purchased from Fischer Scientific, United Kingdom. Potassium dichromate (K_2_Cr_2_O_7_) and ferric chloride hexahydrate (FeCl_3_.6H_2_O) were supplied by BDH Limited Supplies, Poole, England. All solvents were of analytical reagent grade.

### 2.2. Seaweed Species

Brown (*Himanthalia elongata*—sea spaghetti, *Alaria esculenta*—Irish wakame) and red (*Palmaria palmata*—dulse, *Porphyra umbilicalis*—nori) seaweeds harvested off the coast of Co. Clare in Ireland were obtained from a commercial supplier (Wild Irish Seaweed, Co., Clare, Ireland). All seaweeds were 100% naturally grown, organically certified, air-dried (semi-dried), dehumidified and milled prior to purchase. Milled packaged seaweeds were stored at 4 °C until required for analyses. The average particle size of brown and red seaweeds was approximately 2.5 mm [17].

### 2.3. Nutritional Composition

#### 2.3.1. Proximate Composition

Protein (kjeldahl) using nitrogen-to-protein conversion factor of 5 [18], fat (soxhlet with chloroform and methanol (2:1)), moisture (oven at 135 °C for 2 hr) and ash (muffle furnace at 550 °C) contents were analysed by AOAC Methods 954.01, 920.39, 930.15 and 942.05, respectively [19]. Results are expressed as percentages of the dry seaweed weight.

#### 2.3.2. pH and Salt

Seaweeds (5 g) were homogenised for 1 min at 14,000 rpm in 45 mL of distilled water using an Ultra Turrax T25 homogeniser (Janke and Kunkel, IKA-Labortechnik, GmbH and Co., Staufen, Germany). The pH levels of seaweeds were measured at room temperature using a pH meter (Mettler—Toledo, GmbH, Schwerzenbach, Switzerland) by direct insertion of the glass probe.

The salt contents of seaweeds (ashed in a muffle furnace as described in Section 2.3.1) were determined by titration using silver nitrate (AgNO_3_) [20]. The percentage of salt present in seaweed samples was calculated as follows:

(1) $$\%\;\mathrm{salt}\;=\frac{\mathrm{Titre}\;\mathrm{for}\;\mathrm{sample}\left(\mathrm{mL}\right)-\mathrm{Titre}\;\mathrm{for}\;\mathrm{blank}\;\left(\mathrm{mL}\right)}{\mathrm{Mass}\;\mathrm{of}\;\mathrm{sample}\;\left(g\right)}\times {\mathrm{Molarity}\;\mathrm{of}\;\mathrm{AgNO}}_{3}\times 5.844$$

#### 2.3.3. Dietary Fibre

The total dietary fibre (TDF), insoluble dietary fibre (IDF) and soluble dietary fibre (SDF) (AOAC Method 991.43 and AACC Method 32-07.01) contents were determined using an enzyme kit (Megazyme, Bray, Co., Wicklow, Ireland). β-glucan (barley), casein and high-amylose maise starch were used as controls to ensure enzyme purity. Results were expressed as percentages of the dry seaweed weight.

#### 2.3.4. Amino Acid and Mineral Contents

The amino acid profiles and mineral contents of seaweed samples were determined by HPLC with fluorescence detection and pre-column derivatisation (OPA—Ortho Phthalaldehyde derivative), and multi-element, inductively-coupled plasma-emission spectrometry (ICP-OES), respectively. Analysis was carried out by an independent accredited analytical testing facility (ALS laboratories, Little Island, Cork, Ireland). Specific details of the analytical methodologies used by the commercial testing facility were unavailable and confidential.

### 2.4. Total Phenolic Content and In Vitro Antioxidant Activity

#### 2.4.1. Preparation of Polyphenol-Rich Extracts from Ground Seaweeds

Polyphenol rich extracts were prepared as described by Jiménez-Escrig et al. [21] with slight modifications. Brown and red seaweeds (0.5 g) were placed in tubes with 20 mL of methanol/water (50:50). The pH was adjusted to 2.0 using HCl, and tubes were shaken in a water bath (Julabo SW 23, Julabo GmbH, Seelbach, Germany) at 200 rpm for 1 h at 20 °C. Tubes were centrifuged at 4000 rpm for 10 min, and the supernatants were recovered. Acetone/water (70:30) (20 mL) was added to the supernatant residues, and the shaking and centrifugation steps were repeated. Supernatants from both extractions were combined, and extracts were characterised using the total phenol content (TPC), DPPH radical scavenging activity and ferric reducing antioxidant power (FRAP) assays.

#### 2.4.2. Total Phenol Content (TPC)

The total phenol contents of the seaweed extracts were measured using the Folin–Ciocalteu method described by Singleton and Rossi [22] with slight modifications. The extracts of each seaweed were further diluted 1:2 with a mixture of methanol/water (50:50) and acetone/water (70:30), in equal parts, for the assay.

Absorbance measurements were recorded at 750 nm using a UV-vis spectrophotometer (Cary 300 Bio, Varian Instruments, Palo Alto, CA, USA) against a blank containing all reagents and 0.5 mL of the extract solvents. A gallic acid (0.02–0.1 mg/mL) standard curve was prepared in the extract solvents, and results were expressed as milligrams of gallic acid equivalents (GAE)/gram of seaweed.

#### 2.4.3. DPPH Free Radical Scavenging Activity (RSA)

The DPPH free radical scavenging activity of the seaweed extracts was determined as described by Yen and Wu [23] with slight modifications. Absorbance measurements were recorded at 517 nm using a spectrophotometer (Cary 300 Bio) against a methanol blank. An assay blank containing 2 mL of 0.2 mM DPPH and 2 mL of the extract solvents was prepared for calculation purposes. A standard curve of methanolic Trolox (0.01–0.04 mg/mL) was prepared, and results were expressed as milligrams of Trolox equivalent (TE)/gram seaweed.

#### 2.4.4. Ferric Reducing Antioxidant Power (FRAP)

Total antioxidant activity was measured using a modified FRAP method of Benzie and Strain [24] with slight modifications. Absorbance measurements were recorded at 593 nm using a spectrophotometer (Cary 300 Bio) against a blank containing all reagents and 0.15 mL of the extract solvents. A standard curve of methanolic Trolox (0.033–0.1 mg/mL) was prepared, and results were expressed as milligrams of Trolox equivalent (TE)/gram of seaweed.

### 2.5. Thermal Activity

Thermogravimetric analysis (TGA) was carried out using platinum pans on a thermal analyser (Thermal Analysis Q500, TA Instruments, New Castle, DE, USA) using nitrogen gas at a flow rate of 60 mL min^−1^. Seaweed samples (10 mg) were heated at a rate of 10 °C min^−1^ with initial and final temperatures of 35 and 900 °C, respectively. TGA thermograms were analysed using 178 Universal Analysis 2000 software (TA Instruments Ltd., Crawley, United Kingdom), and results are expressed as percentages weight loss as a function of increasing temperature.

### 2.6. Technological Properties

The water holding, oil holding and swelling capacities of seaweeds were measured according to Gómez-Ordóñez et al. [25] with modifications.

#### 2.6.1. Water Holding Capacity (WHC)

Distilled water (20 mL) containing 0.02% sodium azide was added to 0.2 g of seaweed samples in filter paper and stoppered funnels. Samples were allowed to stand for 18 h at 4 °C. Stoppers were removed from the funnels, the residues were weighed and WHC calculated as g of water retained/g of dry sample.

#### 2.6.2. Oil Holding Capacity (OHC)

Commercial virgin olive oil (10 mL) was added to 0.2 g of seaweed samples in 15 mL centrifuge tubes. Samples were stirred using a magnetic stirrer and allowed to stand at room temperature for 18 h. Following centrifugation at 3000× *g* for 20 min, the supernatant was discarded, the residue was weighed and OHC was calculated as g olive oil retained/g of dry sample.

#### 2.6.3. Swelling Capacity (SC)

Seaweed samples (0.5 g) were weighed into 15 mL measuring cylinders (0.1 mL graduations), and 10 mL distilled water containing 0.02% sodium azide was added. Samples were stirred gently to remove air bubbles and placed on a level surface at room temperature for 18 h. Following the 18 h storage period, the volume (mL) occupied by the samples was measured, and SC was expressed as mL occupied/g of dry sample.

### 2.7. Statistical Analysis

Experiments were analysed on three different batches and in triplicate of each batch. The mean of each batch was analysed. Statistical analysis was performed using the IBM SPSS statistics 25 for windows (SPSS, Chicago, IL, USA) software package. One-way ANOVA was used to examine any significant differences between the means at a 5% significance level. Tukey’s post hoc test was used to adjust for multiple comparisons between sample means.

## 3. Results and Discussion

### 3.1. Proximate Composition, Salt Content and pH

Irish wakame possessed higher levels (*p* < 0.05) of protein, fat and salt, and higher pH values compared to sea spaghetti (Table 1). Stévant et al. [26] reported protein (10.2%) and ash (25.6%) contents for Irish wakame comparable to those in the present study. Cofrades et al. [27] reported similar protein (5%), fat (<1.5%), and ash (30%) contents for sea spaghetti. In addition, Fernández-Segovia et al. [28] reported a low protein content of 6.8% for sea spaghetti. A higher level of protein (14.08%) has also been reported previously for sea spaghetti, thus highlighting variability in composition associated with seaweed species [29].

In the red seaweeds, nori contained significantly higher protein levels compared to dulse. However, dulse had higher (*p* < 0.05) moisture, ash, fat, salt contents and pH values than the nori seaweed. Cofrades et al. [27] also reported protein (39%), fat (<1.5%) and ash (12%) contents for nori similar to levels reported in Table 1. By contrast, Taboada et al. [29] reported a higher fat level (2.8%) for nori composed of 26.6% monounsaturated and 26.9% polyunsaturated fatty acids (23.9% w3 and 2.6% w6) and 43.2% saturated fatty acids. In the present study, red seaweeds contained higher protein levels (~19–32%) compared to brown seaweeds (~5.6–10.2%), similar to those observed by Fernández-Segovia et al. [28]. Phycobiliproteins are pigment proteins in red seaweed species, constituting up to 50% of their total protein contents [30]. The high protein contents of red seaweed species are comparable to those of terrestrial plant species consumed as significant sources of protein [29]. For example, red seaweed species such as *Porphyra tenera* (nori) and *Palmaria palmata* (dulse) protein contents have been compared to that of the soybean [31]. The fat contents of seaweeds are typically low (1–3%), and they have PUFA contents similar to those of terrestrial vegetables [31]. However, Gressler et al. [32] reported higher fat contents (3.6% and 6.0%) in red seaweeds (*Plocamium brasiliense* and *Ochtodes secundiramea*, respectively).

The pHs of seaweeds ranged from 5.55 to 6.71 in brown and red seaweed species (Table 1). Abdullah et al. [33] reported pH values of brown seaweed *Sargassum sp.* and red seaweed *E. cottonii* as 6.91 and 6.57, respectively.

### 3.2. Dietary Fibre Content

Total, insoluble (IDF) and soluble (SDF) dietary fibre contents were 31.49–49.13%, 22.02–37.72% and 3.06–12.88%, respectively (Table 2). In the brown seaweeds, sea spaghetti contained higher (*p* < 0.05) TDF and SDF contents than in Irish wakame, though there was no significant difference between their IDF contents. Similar TDF levels for sea spaghetti (50.3%) were observed by Cofrades et al. [27]. However, Gómez-Ordóñez et al. [25] reported higher SDF (23.63%) and lower IDF (13.51%) in Spanish sea spaghetti, indicative of variations in seaweed nutritional composition, which may be due to their maturities, environmental growth conditions, seasonal periods and geographical harvest locations [34]. In red seaweeds, dulse had a higher (*p* < 0.05) SDF content than in nori. However, there were no significant differences in their TDF and IDF contents (Table 2). Cofrades et al. [27] also observed comparable TDF levels for nori (35%). Brown seaweed species contained higher (*p* < 0.05) TDF and IDF compared to red seaweed species, most probably due to the presence of uronic acids from alginate in brown seaweed species, which contribute to the IDF of brown seaweeds [10].

From a nutritional perspective, dietary fibres can increase satiety and aid digestive passage through their bulking capacity [35]. Seaweeds have lower carbohydrate contents than terrestrial foods, the majority of which is dietary fibre. Terrestrial foods contained approximately 1–27% dietary fibre; however, seaweed species can have dietary fibre levels as high as 50–93% [31]. Flynn et al. [36] reported the mean daily intake of dietary fibre in Irish adults as 19 g; however, the recommended intake of dietary fibre is 25–35 g/day [37]. To compare the probable contributions of seaweeds to diet, 8 g DW of seaweed was used as a serving size. Seaweed species examined in this present study, wherein TDF ranged from ~31.4 to 49%, providing 10.5–16.4% of the daily dietary fibre requirement in an 8 g serving.

### 3.3. Amino Acid Content

In the brown seaweeds, Irish wakame had higher (*p* < 0.05) contents of essential amino acids (threonine, valine, iso-leucine and leucine) and all non-essential amino acids, except tyrosine, in comparison to sea spaghetti (Table 3). Irish wakame also had higher (*p* < 0.05) total EAA (essential amino acid), total non-EAA and total AA contents than in sea spaghetti. Total EAA, total non-EAA and total AA contents of Irish wakame were approximately double those of sea spaghetti (Table 3). Cofrades et al. [27] reported similar EAA and total AA contents for sea spaghetti (1.96% and 4.4%, respectively).

Nori had significantly higher contents of essential amino acids (threonine, valine, lysine, iso-leucine, leucine and phenylalanine) and all non-essential amino acids, except cysteine and tyrosine, than dulse. Nori exhibited higher (*p* < 0.05) total EAA, total non-EAA and total AA contents compared to dulse. Total EAA, total non-EAA and total AA contents of nori were also approximately double those of dulse (Table 3). Cofrades et al. [27] also reported comparable EAA and Total AA contents for nori (13.44% and 36.45%, respectively).

Red seaweed species contained a higher (*p* < 0.05) level of each amino acid type (excluding histidine, methionine, phenylalanine, cystine and tyrosine) compared to the brown seaweed species (Table 3). This can be attributed to the significantly higher protein levels present in red seaweed species (Table 1). The total AA contents were comparable with the protein contents of the seaweed species presented in Table 1.

In this study, aspartic and glutamic acids were the most abundantly occurring non-essential amino acids in seaweed species, as observed previously by Gressler et al. [32]. Mabeau et al. [38] reported that the high levels of aspartic and glutamic acids in seaweeds are responsible for their distinct flavour (umami flavour) and taste. Mišurcová et al. [39] also indicated that alanine, glycine and glutamic acid were considered the main components of seaweed flavour.

### 3.4. Mineral Content

Macro (sodium (Na), potassium (K), phosphorus (P), magnesium (Mg) and calcium (Ca)) and trace mineral (iron (Fe), manganese (Mn), zinc (Zn) and copper (Cu)) contents, nutritional intake from an 8 g serving and mineral reference nutrient intake (RNI) levels for children and adults are presented in Table 4. In general, seaweeds contain high levels of minerals due to their capacity to absorb and store inorganic substances from the marine environment [10]. Sea spaghetti had higher Na and Mg contents than Irish wakame; and nori had higher P, Mg, Zn and Cu contents than dulse. Furthermore, brown seaweeds had higher Na, Mg and Ca contents than red seaweed species in this study. Similar findings were reported by Cofrades et al. [27].

Na and K were the most abundant in all seaweed species, consistent with a previously reported study by Cofrades et al. [27]. Na is an essential mineral in the human body; however, excess consumption can be detrimental to human health. A sodium intake of less than 2000 mg per day is recommended [40]. Recently, Morrissey et al. [41] reported a mean dietary sodium intake in Ireland of 2877 mg/day in men and 2134 mg/day in women. Seaweeds contain high concentrations of K (higher than in terrestrial fruits and vegetables), which at high intake levels (≤3510 mg is recommended daily [40]) has been reported to protect against high blood pressure and other cardiovascular diseases [42]. It has been reported that the ratio of sodium to potassium (≤1.0) in the diet may be more significant than the amount of each mineral [43]. The ratio of sodium to potassium (Na:K) in the seaweed species examined was low (Irish wakame—0.74, dulse—0.32 and dulse—0.49), which helps combat fluid retention and high blood pressure without the risk of impacting the potassium balance [44]. However, sea spaghetti had a high Na/K ratio of 1.94, and sources of potassium should be consumed when consuming this seaweed to balance the Na/K ratio.

The phosphorus content of seaweeds ranged from 77.7 to 498 mg/100 g seaweed. This mineral is required in the body in association with calcium. Seaweed species also contained high Mg and Ca contents. Magnesium deficiency in humans is common and has been linked to chronic diseases [45]. Seaweed species in this study can contribute 8.6–26.3% of the Mg RNI of adults in an 8 g serving (Table 4). Seaweeds in this study contained higher Ca levels than present in bovine milk (120 mg/100 g) and spinach (145 mg/100 g) [31].

Dulse had the highest Fe and Mn contents compared to the other trace elements and seaweed species examined (Table 4). MacArtain et al. [1] concluded that specific seaweeds have higher Fe contents than terrestrial sources of Fe, such as spinach and meat. Iron deficiency is one of the most prevalent nutritional deficiencies worldwide in developing and developed nations [46]. Seaweed species in this study can contribute 1.8–27.2% of iron RNI of children and adults in an 8 g serving (Table 4). Mn is also an essential nutrient involved in the processing of cholesterol, carbohydrates and protein in the body. The high Mn levels present in dulse (49.1 mg/100 g) result in the provision of approximately 4 mg Mn in an 8 g serving. This exceeds the safe intake values suggested by the Committee on Medical Aspects of Food and Nutrition Policy [47]; however, a 2.6 g serving will provide approximately 1.3 mg, which is regarded as safe for an adult. The Zn content in seaweed species ranged from 0.94 to 6.12 mg/100 g seaweed. The reference nutrient intake of Cu per day is 1.2 mg (adults), and seaweed species in this study can contribute up to 3% of the RNI in an 8 g serving (Table 4). Other sources of Cu in the human diet include meat and vegetables.

On the other hand, seaweeds can also possess toxic elements such as arsenic (As) and cadmium (Cd), with potentially negative effects on human health. The form of arsenic which is usually of concern is its toxic inorganic form (iAS), which is a known human carcinogen associated with liver, bladder, lung and skin cancers [48]. The majority of arsenic in seaweed appears to be in less toxic forms [49]. Currently, the EU has no limits for arsenic levels in foods; however, the Joint FAO/WHO Expert Committee on Food Additives’ (JECFA) provisional tolerable daily intake (PTDI) for inorganic arsenic is 0.002 mg/kg bodyweight, equivalent to 0.12 mg/day for a 60 kg adult [49]. From previous studies, sea spaghetti, Irish wakame, dulse and nori contain 0.202, 0.220, 0.466 and 0.239 mg/kg of arsenic, respectively [50,51]. The maximum level of cadmium in foods consisting exclusively or mainly of dried seaweed or of products derived from seaweed is 3.0 mg/kg of seaweed or seaweed product [49]. Sea spaghetti, Irish wakame, dulse and nori contain 0.222–0.395, 1.55–2.01, 0.147 and 0.126 mg/kg of cadmium, respectively [50,51].

### 3.5. Total Phenolic Content and In Vitro Antioxidant Activity

**Figure 1:**
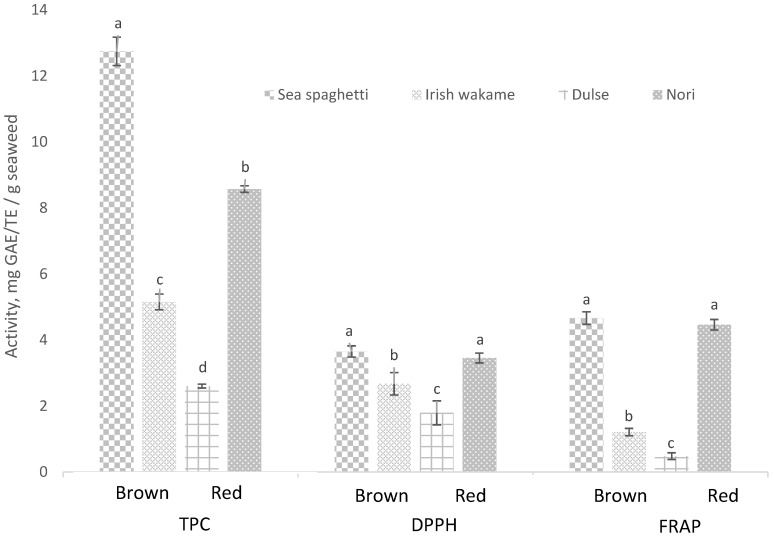
Total phenolic content and in vitro antioxidant activity of brown and red seaweed species. ^abcd^ Mean (±standard deviation) mg GAE/TE/g seaweed. Within each assay, bars containing different letters are significantly different (*p* < 0.05). GAE = gallic acid equivalent, mg/g extract (TPC). TE = Trolox equivalent, mg/g extract (DPPH and FRAP).

A range of compound classes present in seaweeds (carotenoids, phenolic and polyphenolic compounds, pigments, sulphated polysaccharides and vitamins) exhibit varying degrees of antioxidant activity. The level of antioxidant activity or potency associated with a particular seaweed type is influenced by factors such as seasonality, species (red, green or brown seaweeds) and solvents used during the extraction process, i.e., extract manufacturing. In the present study, the total phenolic content (TPC) and in vitro antioxidant activities (DPPH and FRAP) followed the order: sea spaghetti (brown) ≥ nori (red) > Irish wakame (brown) > dulse (red) (*p* < 0.05) (Figure 1).

In brown seaweeds, sea spaghetti had higher (*p* < 0.05) TPC, DPPH and FRAP activities than Irish wakame. Comparable TPC (18 mg GAE/g seaweed) and FRAP activity (4.7–11.7 mg TE/g seaweed) levels in sea spaghetti have been previously reported in the scientific literature [28,52]. Phlorotannins (composed of phloroglucinol sub-units) are unique to brown seaweeds and represent the major class of polyphenolic compounds present. Ummat et al. [53] reported higher TPC, total phlorotannin and flavonoid levels, and DPPH and FRAP activities in sea spaghetti than in Irish wakame, similar to findings reported in the current study (Figure 1). The magnitude of difference between sea spaghetti and Irish wakame DPPH activities was lower than observed for the TPC activity. This may be attributed to the presence of antioxidant compounds such as fucoxanthin, which has been previously reported at higher levels in Irish wakame (0.8–0.9 mg/g) than in sea spaghetti (0.3–0.4 mg/g) [54]. In the absence of a detailed chemical characterisation of the antioxidant compounds present in each seaweed, using, for example, liquid chromatography mass spectrometry analytical techniques, differences in DPPH and FRAP activities were attributed to compositional variations in the profiles of antioxidant compounds contained in the brown seaweed species examined.

In red seaweeds, nori had higher (*p* < 0.05) TPC, DPPH and FRAP activities compared to dulse (Figure 1). Similarly, Ferraces-Casais et al. [55] also reported a higher TPC for nori compared to dulse. Phenolic compound classes present in red seaweeds include bromophenols, flavonoids, phenolics acids, phenolic terpenoids and mycosporine-like amino acids (MAAs) [56]. To date, the scientific literature contains limited information on the identification of such phenolic compounds, including those contained in the red species examined in this study. Polyphenolic flavonoid catechin compounds are present in many seaweed species. Rodríguez-Bernaldo de Quirós et al. [3] reported higher levels of catechins and associated catechin isomers (epicatechin (EC), epigallocatechin (EGC) and epigallocatechin gallate (EGCG)) in nori (*Porphyra* spp.) compared to dulse (*Palmaria* spp.). This may, in part, explain the higher TPC levels observed in the nori seaweed. In addition, Ferraces-Casais et al. [55] reported higher levels of carotenoids (β-carotene, lutein), pigments (chlorophyll a, pheophytin a) and vitamins C (ascorbic acid) and E (tocopherols) in nori compared to dulse, all of which would contribute to the higher (*p* < 0.05) DPPH and FRAP activities observed in the nori species.

### 3.6. Thermal Activity

**Figure 2:**
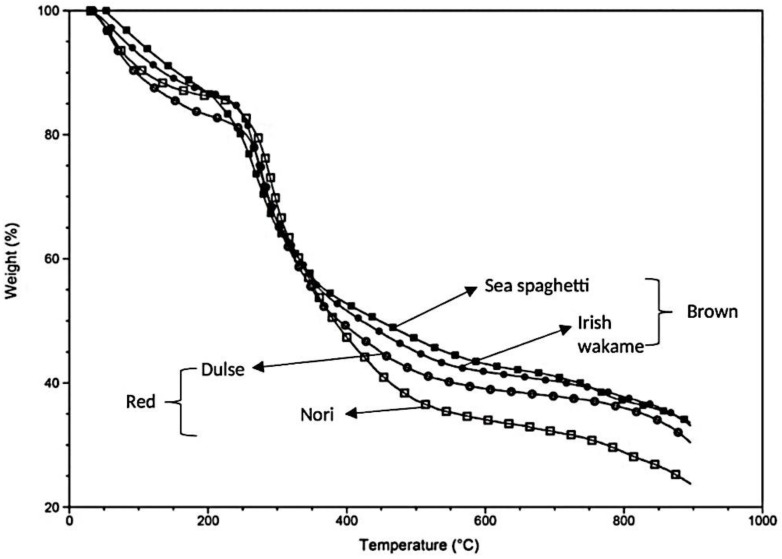
Thermogravimetric analysis curves of brown and red seaweed species under a nitrogen atmosphere.

The TGA curves of brown and red seaweeds are shown in Figure 2. The initial weight loss (35 °C to approximately 200 °C) was due to dehydration (cellular and external water loss). The maximum rate of weight loss occurred at approximately 250 °C. The majority of degradation of components occurred approximately between 200 and 400 °C. Yanik et al. [57] reported the main degradation of seaweeds occurred between 190 and 390 °C and maximum weight loss at 260 °C. Ross et al. [58] also reported that carbohydrate decomposition occurred in a range of 180–270 °C and protein at 320–450 °C. At 550 °C, the mass losses of sea spaghetti and Irish wakame were approximately 55% and 57%, respectively. At 900 °C, approximately 35% of components were present in brown seaweed species, which may decompose at a higher temperature.

At 550 °C, the mass losses of dulse and nori were approximately 60% and 65%, and at 900 °C, about 30% and 24% of components were present in dulse and nori, respectively. Results indicated that organic nutritional components (carbohydrates) in seaweeds and seaweed food products cannot withstand processing temperatures greater than 250 °C. Seaweed composition varies; therefore, the degradation behaviour may also vary.

At 900 °C, the high inorganic residue contents of these seaweed species were possibly due to their high mineral content (Table 4) and other unknown components, which may decompose at a high temperature (>900 °C). These minerals and components may be difficult to pyrolyze and therefore pyrolyze at slow rates—most likely representing constituents of the final weight. Weight loss (%) at 900 °C followed the same trend as the results for ash analysis: sea spaghetti ≥ Irish wakame > dulse > nori (Table 1).

### 3.7. Technological Properties

The water holding capacity (WHC), oil holding capacity (OHC) and swelling capacity (SC) data for brown seaweeds indicated no significant differences (*p* < 0.05) between sea spaghetti and Irish wakame (Table 5). Gómez-Ordóñez et al. [25] reported similar WHC (7.26 g/g dry weight) and OHC (1.61 g/g dry weight), and slightly higher SC (10.97 mL/g dry weight) for sea spaghetti. In the red seaweeds, nori had higher (*p* < 0.05) WHC and SC than dulse. Nori also had higher OHC than dulse, not significantly though (*p* > 0.05). Dulse had lower (*p* < 0.05) WHC and SC than the other seaweeds examined in this study (Table 5). Rupérez and Saura-Calixto [10] reported similar WHC (5.12 g/g dry weight), and lower SC (6.08 mL/g dry weight) and OHC (1.04 g/g dry weight) for nori. In this study, brown seaweed species had lower (*p* < 0.05) OHC than red seaweed species.

The seaweed species examined in this research had high total dietary fibre contents (>30% DW), and those, combined with the protein contents (Table 1), represented approximately 40–55% DW. Wong and Cheung [59] and Yaich et al. [60] suggested that the total dietary fibre, types of polysaccharides (alginate, agar and carrageenan), protein content and proteins present in the cell wall may have an impact on the technological properties (WHC, OHC and SC) of seaweed species. Other than the structure of fibres, technological properties can also be affected by factors such as temperature, particle size, porosity, pH, ionic strength, types of ions in solutions and density are also important to understand the different behaviours of samples during hydration [61]. Factors listed above or a combination of factors may be responsible for the lower WHC in dulse and higher SC in nori. Wong and Cheung [59] and Yaich et al. [60] also proposed that the mechanism of OHC of seaweeds is mainly due to the physical entrapment of oil by capillary attraction and the hydrophobicity of proteins. Additionally, the variation in OHC amongst seaweed species may be due to the different proportions of polar side chains of the amino acids on the surfaces of protein molecules. The red seaweed species examined in this study contained higher protein levels than brown seaweed species (Table 1), which explains their higher capacity to hold oil.

## 4. Conclusions

Brown and red Irish seaweed species represent potential nutritional sources of protein, amino acids, dietary fibre, minerals and health-promoting bioactive antioxidant compounds in the human diet. The seaweed species contained low fat levels, thereby reducing their caloric content when ingested alone or as components of a food product. Brown seaweed species are significant sources of minerals (Na, Mg and Ca), total and insoluble dietary fibres, whereas red seaweed species possessed superiority with respect to their protein content, phosphorus and trace mineral levels.

Sea spaghetti and nori displayed the highest TPC and in vitro antioxidant activity, indicating the suitability of these seaweed species as superior sources of bioactive compounds in the diet or as ingredients for use in the manufacture of functional food products. From a food processing and technological perspective, the seaweeds demonstrated thermal stability at a high temperature and varying degrees of water, oil and swelling capacities, which would ultimately influence the textural properties of food products. Further research is necessary to explore the use of commercially available and sustainable Irish seaweed species as health-promoting functional ingredients in various food systems, such as processed foods, meat products, vegan foods and high protein meals.

## Figures and Tables

**Table 1:** Proximate compositions, salt contents and pHs of brown and red seaweed species.

Seaweed Type		Protein	Moisture	Ash	Fat	Salt	pH
Brown	Sea spaghetti	5.57 ± 0.22 ^a^	16.31 ± 0.34 ^a^	30.90 ± 0.44 ^a^	1.19 ± 0.12 ^ac^	12.68 ± 0.21 ^a^	5.55 ± 0.03 ^a^
	Irish wakame	10.21 ± 0.36 ^b^	11.79 ± 0.12 ^b^	30.06 ± 0.28 ^b^	2.03 ± 0.10 ^b^	15.30 ± 0.32 ^b^	6.29 ± 0.01 ^b^
Red	Dulse	19.04 ± 0.19 ^c^	10.04 ± 0.41 ^c^	25.63 ± 0.67 ^c^	1.52 ± 0.12 ^a^	14.70 ± 0.95 ^ab^	6.71 ± 0.01 ^c^
	Nori	32.03 ± 1.09 ^d^	8.99 ± 0.16 ^d^	17.24 ± 0.22 ^d^	1.02 ± 0.02 ^c^	5.35 ± 0.12 ^c^	6.12 ± 0.01 ^d^

^abcd^ % (DW) Mean values (±standard deviation) in the same column bearing different superscripts are significantly different (*p* < 0.05).

**Table 2:** Make-up of dietary fibre in brown and red seaweed species.

Seaweed Type		TDF	IDF	SDF
Brown	Sea spaghetti	49.13 ± 0.92 ^a^	37.49 ± 0.89 ^a^	11.65 ± 0.38 ^a^
	Irish wakame	40.78 ± 1.57 ^b^	37.72 ± 1.13 ^a^	3.06 ± 1.03 ^b^
Red	Dulse	34.89 ± 2.93 ^c^	22.02 ± 3.33 ^b^	12.88 ± 1.33 ^a^
	Nori	31.49 ± 1.83 ^c^	23.61 ± 1.28 ^b^	7.88 ± 0.56 ^c^

^abc^ % (DW) Mean values (±standard deviation) in the same column bearing different superscripts are significantly different (*p* < 0.05).

**Table 3:** The amino acid contents of brown and red seaweed species.

Amino Acids		Brown Seaweed		Red Seaweed	
		Sea Spaghetti	Irish Wakame	Dulse	Nori
Essential	Histidine	0.09 ± 0.00 ^a^	0.13 ± 0.06 ^a^	0.20 ± 0.15 ^ab^	0.47 ± 0.01 ^b^
	Threonine	0.23 ± 0.00 ^a^	0.50 ± 0.01 ^b^	0.84 ± 0.02 ^c^	1.75 ± 0.09 ^d^
	Valine	0.25 ± 0.01 ^a^	0.53 ± 0.01 ^b^	0.98 ± 0.02 ^c^	1.70 ± 0.08 ^d^
	Methionine	0.14 ± 0.05 ^a^	0.13 ± 0.15 ^a^	0.20 ± 0.23 ^a^	0.29 ± 0.35 ^a^
	Lysine	0.31 ± 0.03 ^a^	0.57 ± 0.02 ^a^	1.19 ± 0.07 ^b^	1.63 ± 0.16 ^c^
	Iso-leucine	0.20 ±0.01 ^a^	0.39 ± 0.00 ^b^	0.62 ± 0.00 ^c^	1.06 ± 0.09 ^d^
	Leucine	0.35 ± 0.01 ^a^	0.74 ± 0.01 ^b^	1.16 ± 0.03 ^c^	2.20 ± 0.00 ^d^
	Phenylalanine	0.30 ± 0.07 ^a^	0.51 ± 0.00^ab^	0.73 ± 0.07 ^b^	1.25 ± 0.06 ^c^
Total EAA		1.86 ± 0.19 ^a^	3.51 ± 0.22 ^b^	5.92 ± 0.31 ^c^	10.36 ± 0.55 ^d^
Non-Essential	Aspartic	0.48 ± 0.02 ^a^	1.11 ± 0.00 ^b^	1.90 ± 0.01 ^c^	3.41 ± 0.00 ^d^
	Serine	0.27 ± 0.00 ^a^	0.56 ± 0.02 ^b^	1.09 ± 0.00 ^c^	1.72 ± 0.01 ^d^
	Glutamic	0.59 ± 0.01 ^a^	1.32 ± 0.15 ^b^	2.01 ± 0.17 ^c^	4.15 ± 0.06 ^d^
	Glycine	0.27 ± 0.01 ^a^	0.59 ±0.01 ^b^	1.13 ± 0.02 ^c^	2.02 ± 0.01 ^d^
	Arginine	0.25 ± 0.01 ^a^	0.49 ± 0.03 ^b^	1.07 ± 0.06 ^c^	3.73 ± 0.09 ^d^
	Alanine	0.33 ± 0.01 ^a^	0.93 ± 0.10 ^b^	1.36 ± 0.02 ^c^	3.90 ± 0.01 ^d^
	Proline	0.20 ± 0.01 ^a^	0.42 ± 0.02 ^b^	0.89 ± 0.10 ^c^	1.30 ± 0.01 ^d^
	Cystine	0.07 ± 0.00 ^a^	0.26 ± 0.00 ^b^	0.34 ± 0.02 ^c^	0.24 ± 0.01 ^b^
	Tyrosine	0.12 ± 0.00 ^a^	0.33 ± 0.00 ^ab^	0.34 ± 0.42 ^ab^	0.99 ± 0.01 ^b^
Total Non-EAA		2.58 ± 0.06 ^a^	6.01 ± 0.33 ^b^	10.13 ± 0.76 ^c^	21.44 ± 0.16 ^d^
Total AA		4.44 ± 0.25 ^a^	9.52 ± 0.55 ^b^	16.05 ± 1.07 ^c^	31.80 ± 0.71 ^d^

^abcd^ % (DW) Mean values (±standard deviation) in the same row bearing different superscripts are significantly different (*p* < 0.05).

**Table 4:** The mineral contents of brown and red seaweed species and RNI of each mineral type.

Minerals		Mineral Levels				Nutritional Intake (8 g Serving)				RNI ^#^	
		Brown Seaweeds		Red Seaweeds		Brown Seaweeds		Red Seaweeds		Infants and Children	Adults
		Sea Spaghetti	Irish Wakame	Dulse	Nori	Sea Spaghetti	Irish Wakame	Dulse	Nori		
Macro	Na	5419 ± 3.61 ^a^	3920 ± 4.73 ^b^	2080 ± 0.29 ^c^	1710 ± 4.86 ^d^	433.60	313.60	166.40	136.80	210–1600	1600
	K	2791 ± 7.21 ^a^	5331 ± 2.65 ^b^	6440 ± 1.80 ^c^	3460 ± 4.75 ^d^	223.20	426.40	515.20	276.80	800–3100	3500
	P	77.70 ± 0.65 ^a^	311 ± 2.31 ^b^	354 ± 0.76 ^c^	498 ± 1.04 ^d^	6.22	24.88	28.32	39.84	270–770	540
	Mg	985 ± 2.50 ^a^	797 ± 1.76 ^b^	322 ± 1.5 ^c^	363 ± 3.51 ^d^	78.80	63.76	25.76	29.04	55–300	270–300
	Ca	957 ± 3.04 ^a^	986 ± 1.53 ^b^	282 ± 3.61 ^c^	152 ± 2.65 ^d^	76.56	78.88	22.56	12.16	525–750	700
Na:K		1.94	0.74	0.32	0.49						
Trace	Fe	1.96 ± 0.03 ^a^	8.74 ± 0.03 ^b^	50.30 ± 0.72 ^c^	20.20 ± 1.04 ^d^	0.16	0.70	4.02	1.62	8.7–14.8	1.7–14.8
	Mn	2.49 ± 0.04 ^a^	0.986 ± 0.00 ^b^	49.13 ± 0.32 ^c^	4.43 ± 0.09 ^d^	0.20	0.08	3.93	0.35	0.016 *	1.4 *
	Zn	0.94 ± 0.00 ^a^	2.21 ± 0.04 ^b^	3.93 ± 0.02 ^c^	6.12 ± 0.03 ^d^	0.08	0.18	0.31	0.49	4.0–9	7.0–9.5
	Cu	0.065 ± 0.01 ^a^	0.109 ± 0.00 ^a^	0.40 ± 0.00 ^b^	0.547 ± 0.06 ^c^	0.005	0.009	0.03	0.04	0.3–1.0	1.2

^abcd^ mg/100 g Mean values (±standard deviation) in the same row bearing different superscripts are significantly different (*p* < 0.05). Na:K ratio—dimensionless. ^#^ RNI—reference nutrient intake in mg/day (Committee on Medical Aspects of Food and Nutrition Policy [47]). * Safe Intakes.

**Table 5:** Technological properties (WHC, OHC and SC) of brown and red seaweeds species.

Seaweed Type		WHC (g Water Held/g Dry Sample)	OHC (g Olive Oil Retained/g Sample)	SC (mL Occupied by Sample/g Dry Sample)
Brown	Sea spaghetti	6.91 ± 0.57 ^a^	1.37 ± 0.05 ^a^	7.50 ± 0.08 ^a^
	Irish wakame	6.69 ± 0.37 ^a^	1.73 ± 0.17 ^a^	7.95 ± 0.13 ^a^
Red	Dulse	3.76 ± 0.23 ^b^	2.90 ± 0.50 ^b^	6.30 ± 0.15 ^b^
	Nori	6.89 ± 0.42 ^a^	3.59 ± 0.13 ^b^	13.22 ± 0.45 ^c^

^abc^ Mean values in the same column bearing different superscripts are significantly different (*p* < 0.05).

## Data Availability

Data is contained within the article.
